# Improvement of Physiological Metabolism and Flavor Quality of *Eriocheir sinensis* Ovaries by Dietary Supplementation with Antarctic Krill Meal

**DOI:** 10.3390/foods14081287

**Published:** 2025-04-08

**Authors:** Siqi Zhou, Renyue Zhang, Zehui Qiu, Yuyao Shi, Shaicheng Zhu, Xugan Wu, Xichang Wang, Long Zhang

**Affiliations:** 1 College of Food Science and Technology, Shanghai Ocean University, Shanghai 201306, China; zhousiqi519@163.com (S.Z.); zhangry1012@163.com (R.Z.); drongker@163.com (Z.Q.); edie_shiyuyao@163.com (Y.S.); xcwang@shou.edu.cn (X.W.); 2 Shanghai Engineering Research Center of Aquatic-Product Processing and Preservation, Shanghai 201306, China; 3 Shanghai Collaborative Innovation Center for Cultivating Elite Breeds and Green-Culture of Aquaculture Animals, Shanghai Ocean University, Shanghai 201306, China; sczhu@shou.edu.cn (S.Z.); xgwu@shou.edu.cn (X.W.); 4 National Demonstration Centre for Experimental Fisheries Science Education, Shanghai Ocean University, Shanghai 201306, China; 5 Centre for Research on Fish Nutrition and Environmental Ecology of the Ministry of Agriculture, Shanghai Ocean University, Shanghai 201306, China

**Keywords:** Antarctic krill meal, *Eriocheir sinensis*, metabolomics, physiological metabolism, thermal reaction pathways, flavor quality

## Abstract

This study investigated the effects of dietary Antarctic krill meal (AKM) on the physiological metabolism and flavor quality of adult *Eriocheir sinensis* ovaries during the postharvest temporary rearing. The AKM concentrations tested were 0% (including negative control group and positive control group), 2%, 4%, 6%, and 8%. The results indicate that the *E. sinensis* ovaries in 8% AKM group produced the highest levels of aroma compounds after thermal processing, including hexanal, heptanal, phenylacetaldehyde, 3-octanone, and 2-methylbutanoic acid ethyl ester. The 8% AKM and negative control group were analyzed by UPLC-MS/MS combined with the nontargeted and widely targeted metabolomics technique. The AKM altered the composition of aroma precursors by adjusting the metabolism of glycerophospholipid, linoleic acid, α-linolenic acid, and amino acid in ovaries. Moreover, lipids composed of polyunsaturated fatty acids (PUFAs) were significantly upregulated (*p* < 0.05). Dietary supplementation with 8% AKM had the best effect on improving the ovarian flavor quality of *E. sinensis*. During the postharvest temporary rearing, more aromatic precursors were produced by regulating physiological metabolism. The ovarian flavor was enhanced by lipid oxidation, Maillard reaction, and Strecker degradation during thermal processing.

## 1. Introduction

With the continuous growth of the global population, aquaculture emerged as a critical pillar of sustainable food production systems, providing nearly 50% of the world’s aquatic animal protein while alleviating pressure on overexploited wild fisheries. Within this context, optimizing the nutritional quality of farmed species through eco-friendly feed strategies becomes imperative to balance productivity with planetary health. Chinese mitten crab (*Eriocheir sinensis*) is a significant economic freshwater aquaculture species, appreciated for its nutritional value and unique flavor. Ovaries are the primary edible tissue of *E. sinensis*, and their odor quality greatly impacts consumers’ purchasing decisions. As aquaculture scales increase and technology advances, exploring the quality improvement technology and mechanism of aquatic product in the aquaculture process has become a growing consensus in the global aquaculture industry [1]. Mixed baits containing small fish, wheat, corn, and soybeans are commonly used during *E. sinensis* culture [2]. The development of environmentally friendly compound feeds that enhance the flavor quality of crabs is imperative.

Antarctic krill (*Euphausia superba*) is a crustacean with the largest biomass found in the Southern Ocean. Due to its high nutritional value, Antarctic krill can also serve as a sustainable source of high-quality protein [3]. Antarctic krill meal (AKM) is processed from Antarctic krill, and its nutritional value is equivalent to that of fish meal [4]. It contains a complete range of amino acids necessary for fish growth, along with abundant polyunsaturated fatty acids (PUFAs), especially eicosapentaenoic acid (EPA) and docosahexaenoic acid (DHA), as well as carotenoids, phospholipids, and minerals [5,6]. These nutrients can stimulate appetite and promote growth in aquatic animals, making AKM an effective substitute for fishmeal and fish oil in feeds [7]. AKM has been used in aquafeeds for European bass and salmon [4,8]. However, research on the improvement of flavor quality in crustacean feed remains limited. Previous studies demonstrated that dietary supplementation with AKM at 5% significantly enhances growth performance, antioxidant capacity, and immune function in crabs [9]. However, research on the improvement of flavor quality in crustacean feed remains limited. Given that the price of Antarctic krill meal is nearly double that of fish meal, feed manufacturers in practical production cannot accept a content exceeding 8%. Considering the cost, the addition of AKM is generally not more than 8%.

Therefore, within the broader context of advancing sustainable aquaculture and meeting rising consumer demand for premium-quality crustacean products, this study aimed to analyze the differences and relationships between the physiological metabolites and flavor quality of *E. sinensis* ovaries with varying concentrations of dietary AKM, exploring the application of AKM in crab feed formulations to improve the flavor quality of *E. sinensis*. Gas chromatography–ion mobility spectrometry (GC-IMS) was utilized to conduct a comparative analysis of volatile components in *E. sinensis* ovaries, allowing for the selection of the optimal aroma quality addition group. Additionally, a nontargeted and widely targeted metabolomics technique based on ultra performance liquid chromatography tandem mass spectrometry (UPLC-MS/MS) was employed to investigate the metabolic differences between the optimal addition group and negative control group for both raw and cooked ovaries, followed by an analysis of differential metabolic and thermal reaction pathways. This study provides fundamental data for the application of AKM in *E. sinensis* feed, which is crucial for developing flavor enhancement strategies to produce high-quality commercial crabs.

## 2. Materials and Methods

### 2.1. Materials

N-ketones (C4–C9), ammonium formate, ammonia, and formic acid were from Aladdin (Shanghai, China). Methanol, acetonitrile, isopropyl alcohol, and methyl tert-butyl ether were purchased from Merck (Darmstadt, Germany). Acetic acid was sourced from Rhawn (Shanghai, China). Dichloromethane was acquired from Thermo Fisher Scientific (Waltham, MA, USA).

### 2.2. Diet Formulation and Preparation

The experimental feed was prepared in a feed mill (Buhler Machinery Co., Ltd., Changzhou, China). Six types of experimental feed were prepared with 0% (including negative control group and positive control group), 2%, 4%, 6%, and 8% AKM. The negative control group and positive control group were designed to investigate whether the experimental groups’ results can surpass the current optimal performance, thereby further enhancing crab quality. All other ingredients were provided by the feed mill. AKM was from QRILL^TM^ Aqua Total of Aker BioMarine (Oslo, Norway). The krill were caught in Statistical Area 48 under the supervision of the Convention for the Conservation of Antarctic Marine Living Resources (CCAMLR). Its main components include protein (58%), fat (25%), astaxanthin esters (115 mg/kg), and omega-3 fatty acids (24%). The protein sources for the basic feed included soybean meal, soy protein concentrate, rapeseed meal, fish meal, chicken meal, peanut meal, gluten meal, chicken meal, and blood meal. Phospholipid oil, fish oil, and soybean oil served as fat sources. Flour was used to adjust energy balance, while soybean oil was utilized to balance total fat content. To enhance feed stability in water, gluten powder acted as an adhesive, and some fats were applied using a vacuum spray. These feed formulas are designated as negative control group, 0% (NG), and positive control group, 0% (PG), 2%, 4%, 6%, and 8%. The formulation for each experimental diet is shown in Table 1.

### 2.3. Experimental Design and Feeding Trials

The experimental crabs were taken from the Chongming Research Station of Shanghai Ocean University and the breeding trials were conducted there. The rearing experiment was conducted in 18 polyethylene tanks (Φ = 1.08 m, H = 1.20 m) with three replicates (*n* = 180). Ten female *E. sinensis* were randomly put in a tank after a seven-day reproductive molt (individual body weight: 70–80 g). Black plastic shelters were placed at the bottom of the tanks, and the water depth was consistently maintained at approximately 50 cm. During the culture period, crabs received their respective diets daily at 6 PM. Water quality indicators were measured every 3 days and necessary adjustments or water additions were made every 2 weeks based on the obtained water quality indicators. Water quality requirements were as follows: pH: 7.0–9.0; dissolved oxygen: >4 mg L^−1^; ammonia nitrogen: <0.5 mg L^−1^; and nitrite: <0.15 mg L^−1^. The feeding experiment period was 70 days.

### 2.4. Ovarian Tissue Collection

All animal experiments were approved by the Experimental Animal Ethical Review Committee of Shanghai Ocean University (No. SHOU-DW-2022-069). Six female crabs were randomly selected from each tank after a 24 h fasting period at the end of a 70-day fattening period. Ovarian tissue was manually separated from crab body with pliers at a temperature of 4–7 °C. To prevent the crabs from struggling and to avoid unnecessary pain, anatomical needles were inserted into the crab mouth to quickly sever the nerves along the abdomen. The carapace was detached from the crab body, after which the ovaries were carefully collected from the carapace using tweezers [1]. The sample (1.00 g) was placed in a glass vial, then heated in a 100 °C water bath for 10 min. The whole crab is usually steamed at 100 °C for 20 min by consumers. In this study, steaming was performed on isolated ovaries under 100 °C for 10 min to mimic conventional thermal processing. The raw and cooked samples were divided into negative control raw ovary (NR), negative control cooked ovary (NC), experimental raw ovary (ER), and experimental cooked ovary (EC). Samples were then stored at −80 °C for biochemical analyses.

### 2.5. GC-IMS Analysis of Volatile Composition

Each sample was weighed at 1.00 g and put into a 20 mL headspace bottle, then incubated at 100 °C for 20 min prior to injection. The injection volume was 500 μL, and the injection needle temperature was 85 °C. Three parallel determinations were determined for each sample using GC-IMS (FlavourSpec^®^ sensitive analyzer, G.A.S., Dortmund, Germany). The gas chromatography (GC) parameters included a column temperature that was set to 60 °C, and a MXT-WAX capillary column (Restek, USA) was used. Nitrogen with 99.99% purity served as the carrier gas, and the operation time was 20 min. The programmed inlet gas flow was as follows: the first 2 min, flow rate at 2.0 mL/min, 2–10 min; 10.0 mL/min, 10–20 min, and finally increased to 100.0 mL/min and maintained for 30 min. Volatile organic compounds (VOCs) were analyzed by VOCal-0.1.1 [gamma] software.

### 2.6. Nontargeted and Widely Targeted Metabolomics Analysis

#### 2.6.1. Metabolome Extraction

##### Extraction of Hydrophilic Metabolites

Samples (20 mg) were removed from the −80 °C refrigerator and homogenized for 20 s in a ball mill (30 Hz). Each group had three biological duplicate samples, and each sample included three individual crabs. Subsequently, they were centrifuged at 3000× *g* for 30 s at 4 °C. Hydrophilic compounds were extracted by shaking with 400 μL of 70% methanol aqueous internal standard extractant (including 1 mg/L of [2H3]-L-Carnitine HCl, [2H5]-kynurenic acid, [2H5]-hippuric acid, [2H5]-phenoxy acetic acid, L-phenylalanine (HPLC grade, ISOREAG Biotechnology Co., LTD, Shanghai, China), 4-fluoro-L-α-phenylglycine (Shanghai Chemical Industry Development Co., LTD, Shanghai, China), and L-2-chlorophenylalanine (HPLC grade, J&K Scientific, Beijing, China). Samples were centrifuged at 12,000× *g* for 10 min at 4 °C, and 300 µL of the supernatant was shifted to another numbered centrifuge tube and cooled for 30 min at −20 °C. The sample was centrifuged again at 12,000× *g* for 3 min at 4 °C, and 200 µL of the supernatant was transferred to the corresponding liner tube of the injection bottle for metabolomics analysis.

##### Extraction of Hydrophobic Metabolites

Samples were weighed (20 mg) and hydrophobic compounds were extracted by adding 1 mL of internal standard lipid extract (tert-butyl methyl ether: methanol = 3:1, V/V, HPLC grade, Merck, Darmstadt, Germany) to the sample. A total of 200 μL of water was added and vortexed for 1 min, then centrifuged at 12,000× *g* for 10 min at 4 °C. The supernatant (200 mL) was pipetted into the corresponding numbered centrifuge tubes and concentrated until completely dry. The 200 μL of lipid complex solution (acetonitrile: isopropanol = 1:1, V/V, HPLC grade, Merck, Darmstadt, Germany) was added, vortexed for 3 min, and centrifuged at 12,000× *g* for 3 min. The supernatant was prepared for LC-MS/MS analysis.

#### 2.6.2. Chromatography and Mass Spectrometry Acquisition Conditions

##### Analysis Conditions for Hydrophilic Substances

The UPLC-MS/MS system (UPLC, ExionLC AD; MS, QTRAP) was used. Analytical conditions: a T3 column was used, as well as the mobile phases including solvent A (ultrapure water with 0.1% formic acid) and B (acetonitrile with 0.1% formic acid). Sample detections were conducted using a gradient program, and the initial ratio of mobile phase was 95% A and 5% B. Linear elution procedure: 0–2 min, 80% A and 20% B; 3–5 min, 40% A and 60% B; 5–7.5 min, 1% A and 99% B; and 7.6–10 min, 95% A and 5% B. The flow velocity was set as 0.4 mL/min, the column temperature was 40 °C, and the injection volumes were 2 μL (widely targeting assay) and 5 μL (non-targeting assay). A HILIC column was used, and the mobile phase contained solvent A (20 mM ammonium formate, 10% methanol, 30% water, and 60% acetonitrile, with pH adjusted to 10.6 with ammonia) and B (20 mM ammonium formate, 40% acetonitrile, and 60% water, with pH adjusted to 10.6 with ammonia). The initial ratio of the mobile phase was 95% A and 5% B. The gradient was programmed to 70% A and 30% B in 3.5 min. After 2 min, the gradient was adjusted to 5% A and 95% B and held for 1 min, returning to 95% A and 5% B and held for 3.5 min. The column temperature, flow rate, and injection volume were the same as those of the T3 column.

UPLC (ExionLC AD) and quadrupole time of flight mass spectrometry (TripleTOF 6600, AB SCIEX Pte. Ltd. Marsiling, Singapore) were used for nontargeted detection. Analysis conditions: temperature: 500 °C; contain gas: 35 psi; ion source gas1: 50 psi; ion source gas2: 60 psi; and IonSpray voltage: −4000~5000 V. The acquisition time was 10 min.

UPLC and tandem mass spectrometry (MS/MS) (QTRAP^®^) were used for widely targeting detection. Conditions: ESI temperature: 500 °C; contain gas: 25 psi; ion source gas1 and gas2: 50 psi; IonSpray voltage: −4500~5000 V; collision-activated dissociation was set to high; and acquisition time was 10 min.

##### Analysis Conditions for Hydrophobic Substances

The UPLC-MS/MS system was used. Analytical conditions: A C30 column (Thermo Fisher Scientific, America) was used with mobile phases that consisted of solvent A (10 mM ammonium formate, 40% water, 60% acetonitrile, and 0.1% formic acid) and solvent B (10 mM ammonium formate, 10% acetonitrile, 90% isopropanol, and 0.1% formic acid). Sample measurements were conducted using a gradient program, starting with 80% A and 20% B. The linear elution procedure was as follows: 0–2 min, 70% A and 30% B; 2–4 min, 40% A and 60% B; 4–9 min, 15% A and 85% B; 9–14 min, 10% A and 90% B; 14–17.3 min, 5% A and 95% B; and 17.3–20 min, 80% A and 20% B. The column temperature, flow rate, and injection volume were the same as those of the T3 column. Widely targeted analysis conditions are consistent with those of hydrophilic compounds.

#### 2.6.3. Identification of Differential Metabolites

All sample extracts were mixed in equal amounts to form QC samples, and nontargeted detection was performed on the LC-QTOF-MS/MS platform. Accurate qualitative analysis was performed based on the standard database MWDB (including secondary spectra and retention time RT), DB all public database (including Metlin, HMDB, KEGG, etc.), and MetDNA. Finally, in all samples, precise MRM quantification of metabolites was performed based on the Q-Trap instrument platform.

### 2.7. Statistical Analysis

Statistical analyses of the *E. sinensis* ovary data were conducted using SPSS statistical software version 24.0, with a level of *p* < 0.05. The analysis of variance was employed to ascertain the significance between the means of the distinct samples (*n* = 3). The Waller–Duncan procedure revealed notable discrepancies. Origin 2021 was used to plot bar charts. Statistical analyses such as PCA, orthogonal partial least squares discriminant analysis (OPLS-DA), heat map, and cluster were performed using MetaboAnalyst 5. Cluster correlation heatmaps of important metabolites (variable importance in projection (VIP) >1.5) were plotted using the OmicStudio 19.4 tool.

## 3. Results and Discussion

### 3.1. Analysis of Ovarian Volatile Odor Components

**Figure 1:**
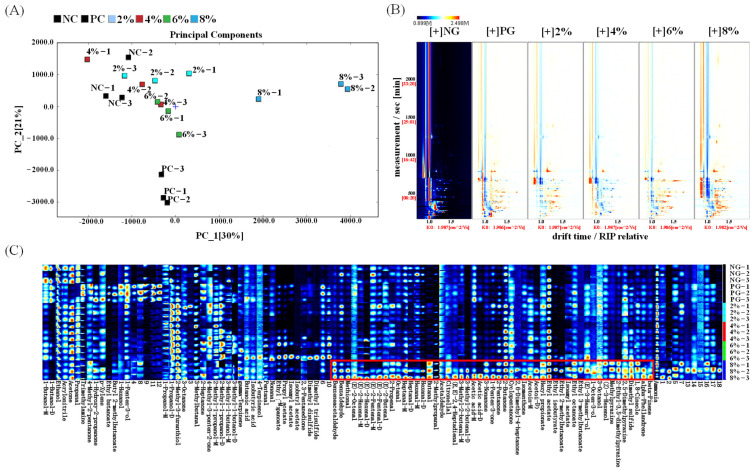
Analysis of volatile compounds in *E. sinensis* ovaries from different AKM addition groups using GC-IMS. (**A**) PCA plot; (**B**) differential comparative chromatogram; and (**C**) fingerprint spectra. NG: negative control group; PG: positive control group; 2%: 2% AKM addition level; 4%: 4% AKM addition level; 6%: 6% AKM addition level; and 8%: 8% AKM addition level.

The PCA results of volatile compounds of *E. sinensis* ovaries from different AKM addition groups are shown in Figure 1A. The two principal components combined accounted for 51% of the variance. The sample distribution between the 8% group and other groups is relatively large, indicating that the 8% group of AKM has a significant impact on the odor of the *E. sinensis* ovaries. There are overlapping areas in the distribution of samples in the NC, 2%, 4%, and 6% groups, indicating similar odor quality among these groups. The spectra of the NC group were selected as controls to obtain the different comparison graphs of the samples (Figure 1B). Compared with other groups, the 8% group showed the greatest change in the content of volatile flavor compounds in both figures. Ninety known volatile compounds were detected in all groups, including 25 aldehydes, 14 ketones, 15 esters, 18 alcohols, etc. From the fingerprint spectrum (Figure 1C), it can be more clearly seen that the control group (NC and PC) had the lowest content of volatile odor substances, and when the content of AKM reached 8%, the content of flavor substances was at the highest level. The contents of substances such as hexanal, heptanal, phenylacetaldehyde, 3-octanone, ethyl 2-methylbutyrate, acetic acid, etc., in the red box are obviously higher than those in other groups, declaring that the addition of AKM helps to improve the flavor of crab ovaries.

Aldehydes have the characteristics of high content and low threshold, and they contribute the most to the flavor of *E. sinensis* [10]. Among the 25 aldehyde substances detected, except for 2-acrylaldehyde, which produces an unpleasant odor, all other aldehydes are aroma components. Some branched aldehydes and phenylaldehydes are well-known aroma compounds, such as acetaldehyde, 2-methylbutanal, phenylacetaldehyde, and benzaldehyde. Benzaldehyde and phenylacetaldehyde are also the main sources of nutty almond flavor, which may be formed by Streker degradation of leucine during the Maillard reaction, or by thermal degradation of carotenoids [11]. The main odor contributors of the *E. sinensis* ovaries include nonanal, octanal, heptanal, and propanal, which have fruity aromas. Hexanal contributes to the grassy and fatty flavors of the ovaries, primarily produced by oxidation of linoleic acid [12]. In addition, the aldehydes mentioned above have a synergistic effect and exhibit strong flavor even under trace conditions, which contributes to the flavor formation of *E. sinensis* ovaries [13].

Esters are usually produced by the esterification reaction of carboxylic acids and alcohols produced by lipid metabolism. Esters provide sweetness, cream, fruit, and floral fragrance, while enhancing the aroma, therefore making significant contributions to the formation of crab aroma. The 8% group contains most of the esters, mainly including methyl acetate, ethyl butyrate, ethyl acetate, ethyl isobutyrate, isobutyl acetate, etc. Alcohols typically produce a softer aroma with fruity or herbal notes. The 8% group contains more alcohols, including trans-3-hexen-1-ol, 1-octen-3-ol, 3-octanol, cis-3-hexen-1-ol, isoamyl alcohol, acetylmethyl methanol, etc. 1-octen-3-ol can produce an ideal fishy odor, which is believed to be the reason for the grassy aroma of crab meat, mainly derived from the degradation products of linoleic acid hydroperoxide [14]. Unsaturated alcohols typically have lower thresholds than saturated alcohols and may have a greater impact on overall flavor [15]. However, Liu believed that 1-octen-3-ol and n-hexanol are the causes of the unpleasant odor. Therefore, 1-octen-3-ol may have a negative effect on the flavor of ovaries [16].

Trimethylamine, which has a negative odor of fish and amine, is an important component that affects the overall aroma characteristics of *E. sinensis* ovaries. The fingerprint spectrum shows that the PG group contains the highest amount of trimethylamine. Dimethyl trisulfide exhibits a putrid or odorous property, with the highest content in the 6% group. It was clear that the quality difference between the NG group and the 8% AKM group was more obvious. Therefore, based on odor quality, the 8% group and the NG group were selected as the experimental group for the subsequent metabolomics analysis to explore the physiological metabolic effects of 8% AKM on the *E. sinensis* ovaries, establish the relationship between physiological metabolism and odor quality, and investigate the mechanism of aroma formation.

### 3.2. Multivariate Statistical Analysis of Metabolites

**Figure 2:**
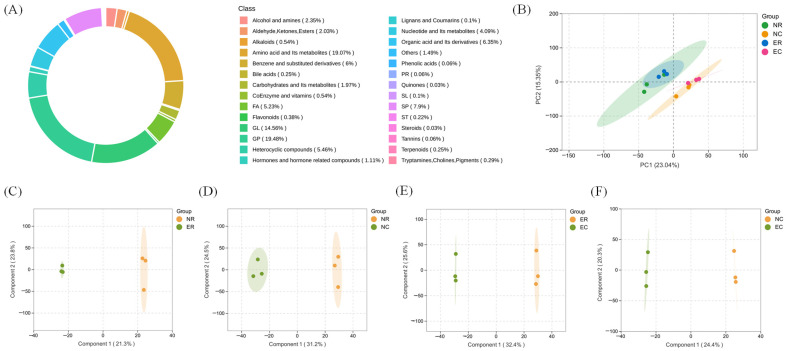
Multivariate statistical analysis of *E. sinensis* ovarian samples with different AKM addition groups. (**A**) Ring chart of categories of metabolites; (**B**) mass spectrometry data set PCA score plot; (**C**–**F**) the orthogonal partial least squares discriminant analysis (OPLS-DA) score plots, (**C**) ER vs. NR; (**D**) NC vs. NR; (**E**) EC vs. ER; (**F**) EC vs. NC. NR: negative control raw ovary; NC: negative control cooked ovary; ER: experimental raw ovary; and EC: experimental cooked ovary.

Metabolomics analysis (Figure 2) was performed by UHPLC-Q-TOF-MS on ovarian samples from the negative control (0% added) and experimental (8% added) groups to analyze the differences of four comparative groups, include ER vs. NR (Group 1), NC vs. NR (Group 2), EC vs. ER (Group 3), and EC vs. NC (Group 4). A total of 3152 metabolites were identified, representing the following classes: glycerophospholipids (19.48%), amino acid and its metabolites (19.07%), glycerolipids (14.56%), sphingolipids (7.9%), organic acid and its derivatives (6.35%), heterocyclic compounds (5.46%), fatty acyls (5.23%), and the other 14 classes of compounds (Figure 2A). According to principal component analysis (PCA) (Figure 2B), the two principal components account for 38.39% of the total variance, and there is a clear separation trend between the raw and cooked samples. The OPLS-DA model with a higher fitting degree was used to visualize the changes in the ovarian metabolomics of the *E. sinensis* fed with different amounts of AKM (Figure 2C–F). Significant separation was observed between sample groups, indicating that dietary AKM and heating treatment caused significant changes in ovarian metabolite levels.

### 3.3. Metabonomic Analysis Based on Group 1 (ER vs. NR)

#### 3.3.1. Screening of Differential Metabolites

**Figure 3:**
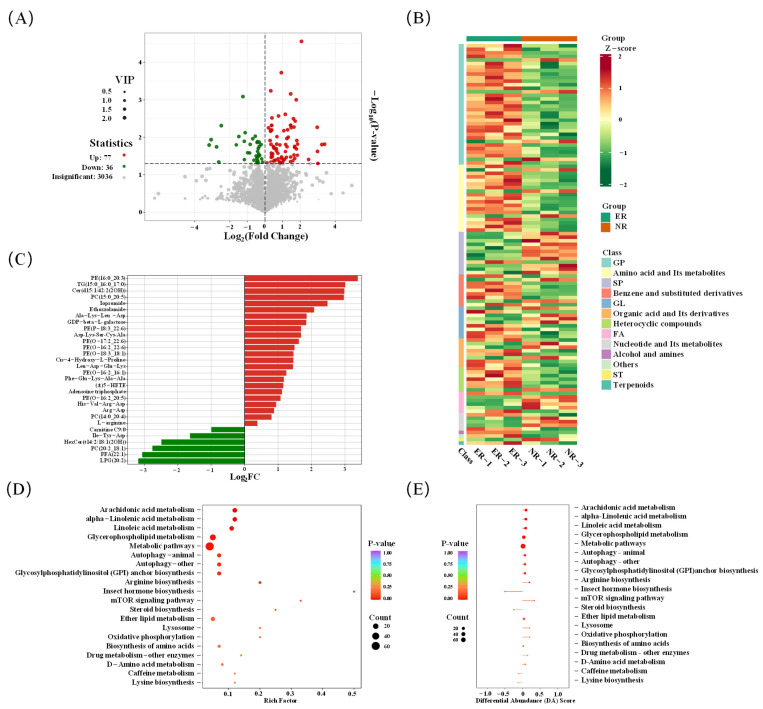
Differential metabolite (DMs) analysis of *E. sinensis* ovaries in Group 1 (VIP > 1, *p* < 0.05). (**A**) Volcano plot; (**B**) cluster heatmap; (**C**) bar chart; (**D**) the top 20 Kyoto Encyclopedia of Genes and Genomes (KEGG) enrichment pathways; and (**E**) differential abundance score chart. Group 1: ER vs. NR; ER: experimental raw ovary; and NR: negative control raw ovary.

The thresholds of VIP value ≥ 1 and *p* value < 0.05 were used for screening differential metabolites. A total of 113 differential metabolites were identified in Group 1 (ER vs. NR) (Figure 3A), of which 77 were upregulated and 36 were downregulated in ER group. From the clustering heatmap (Figure 3B), it can be seen that the content of glycerophospholipids, amino acids and its metabolites, benzene and its derivatives, heterocyclic compounds, etc., in the ER group was significantly higher than that in NR group. Fold change (FC) was used to further screen for differential metabolites, Figure 3C shows the top 30 metabolites with FC, including upregulated PE (16:0_20:3), TG (15:0_16:0_17:0), Cer (d15:1/42:2 (2OH)), PC (15:0_20:5), etc., as well as downregulated LPG (20:2), FFA (22:1), PC (20:2_18:1), Ile-Tyr-Asp, etc. The results show that 8% AKM had a significant impact on the metabolite content in the *E. sinensis* ovaries. These differential metabolites synergistically regulate the metabolic distribution, antioxidant defense, and endocrine system of *E sinensis*, and ultimately promote the absorption and accumulation of amino acids, lipids, and other metabolites in the ovary, leading to upregulation.

#### 3.3.2. Analysis of Lipid in Group 1

The detected lipids mainly include glycerolipids (GL), glycerophospholipids (GP), sphingolipids (SP), and fatty acyls (FA). These lipids can be further divided into triglycerides (TG), diglycerides (DG), phosphatidylcholine (PC), phosphatidylethanolamine (PE), ceramide (Cer), acylcarnitines (CAR), and free fatty acids (FFA). Among them, phospholipids (PL), TG, and FFA contribute the most to aroma [17].

All differential lipids composed of C20:5n-3 (EPA) and C22:6n-3 (DHA) fatty acids in the ER group, such as PC (15:0_20:5) and PE (P-18:3_22:6; O-17:2_22:6), were upregulated (Figure 3C). In addition, most differentially expressed lipids consist of C16:1n-9 (palmitoleic acid) and C18:1n-9 (oleic acid), such as PE(O-18:3_18:1) and PE(O-16:2_16:1), were significantly downregulated. DHA and EPA are PUFAs that are abundant in AKM, mainly in the form of phospholipids, which can promote the development of *E. sinensis* ovaries and are also essential fatty acids for the human body. Most of the EPA and DHA in the metabolic process of *E. sinensis* fed with AKM is esterified into PC, which is present as bound lipids in the ovaries, leading to an increase in the lipid content composed of EPA and DHA in the ER group. Mono/polyunsaturated fatty acids, as volatile flavor precursors, contain a large number of oxidation sites, which can produce various aroma compounds, such as aldehydes and alcohols, after thermal processing. Aldehydes are considered important aroma compounds in *E. sinensis*, and n-3 PUFAs are precursors of (E, E)-2,4-heptadienal, which continuously increase during the heating process [18]. Additionally, (E, E)-2,4-heptadienal with a low odor threshold has sweet, hazelnut, woody, and beef aromas [19]. Consistent with the GC-IMS results, the content of (E, E)-2,4-heptadienal was higher in the 8% group than in the NG group. C16:1n-9 and C18:1n-9 are also abundant in AKM, leading to upregulation of the above-mentioned lipids in ER ovaries [20]. Oleic acid is a monounsaturated fatty acid that can produce volatile flavors, such as 2-heptanone (fruity), 1-hexanol (fruity), and nonanal (floral and fruity), during thermal processing. It is expected that these aromatic compounds will appeal to target consumers due to their specific sensory attributes, e.g., ’harmonious balance of fruity and floral notes’. The increase in lipid content composed of C18:1n-9 provides more precursors for the formation of volatile odor compounds during thermal processing, which led to an increase in the content of the volatile substances mentioned above and affected the overall odor characteristics of the ovaries. Therefore, changes in lipid content in the ER group can affect the flavor of *E. sinensis* ovaries [20]. It is suggested that the downregulation of PC (20:2_18:1) is due to the increased involvement of C18:1n-9 in the synthesis of other lipids, leading to upregulation of lipids composed of C18:1n-9. Since C14:0 and C16:0 (palmitic acid) are the most abundant saturated fatty acids (SFA) in AKM, most of the lipids in ER that consist of the above-mentioned fatty acids, such as PC (14:0_20:4) and PE (16:0_20:3), tended to be upregulated after lipid metabolism. In addition, cholesterol was found to be downregulated in the ER group, which is an essential nutrient for aquatic animals and has significant implications for ovarian development. Cholesterol, as a precursor for the synthesis of steroid hormones, is transported to the mitochondria. After a series of complex enzymatic reactions, it is gradually converted into steroid hormones, which participate in the regulation of the synthesis of vitellogenin in crustaceans [21]. One of the main changes during the maturation of crustacean oocytes is the accumulation of vitellin. As a precursor molecule of vitellin, vitellogenin is an important indicator of ovarian development and is transported to the developing oocytes through the hemolymph. Eventually, it accumulates in the oocytes in the form of yolk substances and promotes the development of the ovary [22]. The role of phospholipids rich in n-3 PUFA in reducing cholesterol in AKM has been confirmed, leading to a downregulation of cholesterol in the ER group [23]. Therefore, dietary lipid composition can affect the lipid category and fatty acid composition of aquatic products, thereby altering their odor quality, which has been validated in *E. sinensis*.

#### 3.3.3. Analysis of Amino Acids and Nucleotides

Three free amino acids (FAA) were identified in the ER group, including upregulated L-arginine and DL-arginine (Figure 3C). The AKM contains abundant amounts of glutamic acid and arginine, but no changes in glutamic acid content were detected in differential metabolites [3]. Glutamic acid is a precursor for the biosynthesis of L-arginine, which can be converted into arginine through the urea cycle after decarboxylation to produce ornithine [24]. It may also be due to the presence of L-arginine in AKM, thereby increasing its levels in the ovaries. It was worth noting that although L-arginine is a bitter amino acid, it has been proved that it can enhance the umami and mouthfeel of *E. sinensis*, and it has a significant impact on the overall taste of aquatic products [25]. In addition, studies have shown that adding 2.7–3.7% L-arginine to feed can improve the growth, survival rate, antioxidant capacity, immunity, and disease resistance of juvenile *E. sinensis* [26]. A significant upregulation of adenosine triphosphate (ATP) was detected in the ER group (Figure 3C). Nucleotides play a role in providing energy in living organisms and providing flavor in food. ATP, as a flavor precursor, can be broken down into flavor nucleotides such as IMP, AMP, and GMP during thermal processing [27]. AMP is the main flavor-producing nucleotide accumulated in crab meat, which contributes significantly to the umami taste [28]. Moreover, umami amino acids and flavor nucleotides could produce a synergistic effect in enhancing umami quality. Interestingly, the initial accumulation of ATP-related compounds in organisms determines the final content of flavor nucleotides in cooked meat [29]. Therefore, the addition of AKM in feed can enhance the accumulation of ATP content in *E. sinensis* ovaries and have a positive impact on the umami quality of *E. sinensis* ovaries after thermal processing.

#### 3.3.4. Enrichment Analysis of KEGG Metabolic Pathway

Key metabolites were enriched through the Kyoto Encyclopedia of Genes and Genomes (KEGG) pathway (Figure 3D). The main differential metabolic pathways included α-linolenic acid metabolism, linoleic acid metabolism, glycerophospholipid metabolism, etc. Among them, the most significant changes occurred in the glycerophospholipid metabolic pathway, involving 25 differential metabolites. In addition, the differential metabolites enriched in these four metabolic pathways showed an upregulation trend (Figure 3E). The metabolism of glycerophospholipids increased the content of aroma compounds in abalone muscle and improved flavor quality by regulating the lipid composition in abalone muscle [30]. Cui found that glycerophospholipid metabolism played an important role in the generation of volatile compounds in mackerel during dry curing [31]. The dietary AKM has the greatest impact on lipid metabolism in *E. sinensis* ovaries. Lipids are precursors of many odorants, and the changes in ovarian lipids before and after thermal processing need to be closely monitored.

### 3.4. Analysis of Thermal Reaction Pathways Based on Group 2 (NC vs. NR) and Group 3 (EC vs. ER)

**Figure 4:**
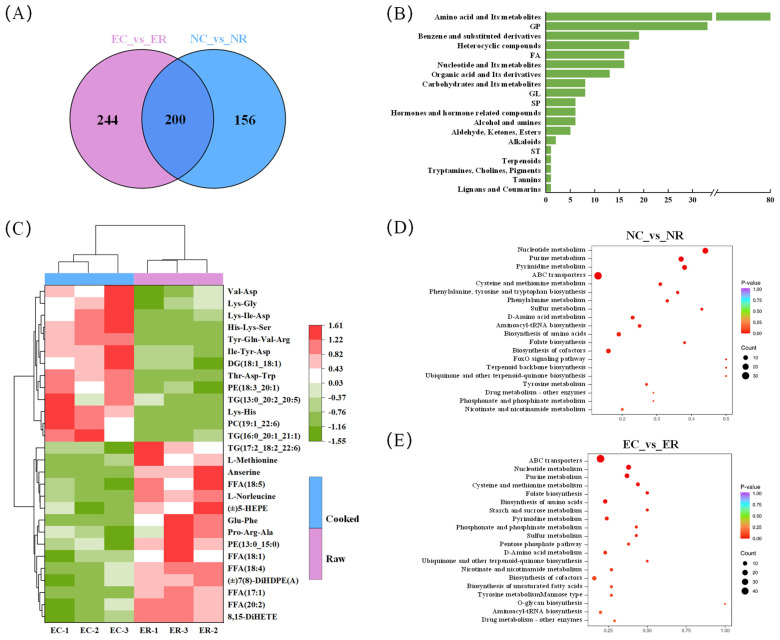
Differential metabolites (DMs) analysis of *E. sinensis* ovaries in Group 2 and Group 3 (VIP ≥ 1, *p* < 0.05). (**A**) Venn diagram; (**B**) bar chart; (**C**) cluster heatmap; and (**D**,**E**) Kyoto Encyclopedia of Genes and Genomes (KEGG) analysis. Group 2: NC vs. NR; Group 3: EC vs. ER; NR: negative control raw ovary; NC: negative control cooked ovary; ER: experimental raw ovary; and EC: experimental cooked ovary.

**Figure 5:**
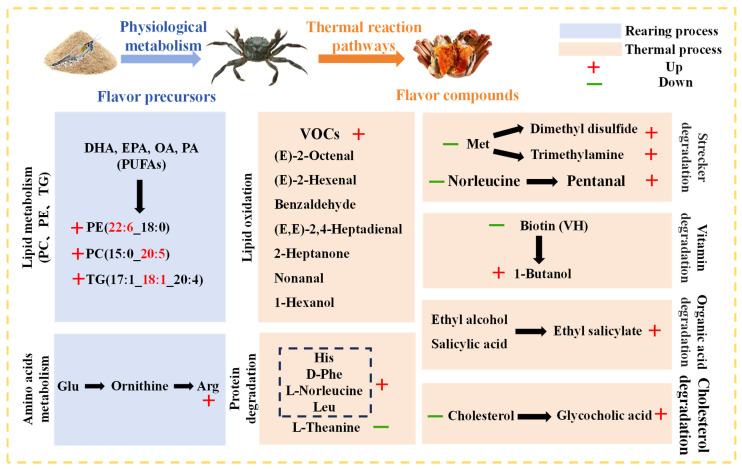
Metabolic pathways of key aroma precursors from *E. sinensis* ovarian during cultivation and thermal processing.

Compounds with significant differences between EC and ER were screened under conditions of VIP value ≥ 1 and *p* value < 0.05. The differential metabolites of Group 3 (EC vs. ER) were compared with those of Group 2 (NC vs. NR) in the Venn diagram (Figure 4A). Since the increase in aroma substances in Group 3 resulted from differential metabolites, the 244 substances in the left area of the Venn diagram are most related to the enhancement of aroma quality. As shown in Figure 4B, 18 types of differential metabolites were only present in Group 3, including 19 benzene and its derivatives, 17 heterocyclic compounds, 16 nucleotide and its metabolites, 16 fatty acyls, 13 organic acid and its derivatives, 8 glycerides, 8 carbohydrates and their metabolites, etc. The heat map (Figure 4C) show that there were significant differences between EC and ER, and the main compounds were FFAs, PE, TG, oxidized lipids, and FAAs. The changes in these compounds were discussed in detail. The differential metabolites were enriched and analyzed by the KEGG analysis platform (Figure 4D,E). The results show that the effects of thermal processing on ovarian metabolic pathways mainly focus on nucleotide metabolism, purine metabolism, and pyrimidine metabolism. The thermochemical reaction during thermal processing can convert flavor precursors in aquatic products into volatile flavor compounds. Figure 5 summarizes the analysis results showing the metabolic pathways of key flavor precursors in the physiological metabolism and thermal processing under the addition of 8% AKM.

#### 3.4.1. Analysis of Lipid

Key lipids downregulated in Figure 4C, such as TG (17:2_18:2_22:6), PE (13:0_15:0), FFA (17:1; 18:1; 18:4; 18:5; 20:2), (±)5-HEPE, 8,15-DiHETE, and (±)7(8)-DiHDPE(A), can be served as important precursors for volatile flavor compounds. In contrast, TG (16:0_20:1_21:1), TG (13:0_20:2_20:5), DG (18:1_18:1), PE (18:3_20:1), and PC (19:1_22:6) were significantly upregulated. These lipids were only contained in Group 3, which was consistent with the above conclusion: DHA (22:6n-3), FFA (18:1), and FFA (18:4) are rich in AKM, so the differential lipids composed of them were only detected in Group 3. The alterations in the content of these lipids were attributable to the structural modifications of lipids such as glycerides or phospholipids during thermal processing, which were hydrolyzed to FFAs, resulting in an increase in the content of FFAs. Part of the FFAs was oxidized and degraded to produce different volatile flavor compounds, resulting in the reduction in lipid content. Moreover, under the action of different lipases, triglycerides were broken down into fatty acids, diacylglycerols, monoglycerides, and glycerols. However, due to the oxidation reaction during thermal processing, the internal chemical bonds of these molecules were reconstituted to form TGs, which led to the increase in TG (16:0_20:1_21:1) and TG (13:0_20:2_20:5) content. In addition, TGs and PLs can also be mutually transformed, resulting in the change in TG content [32].

It was reported that aldehydes and alkanes, such as (E)-2-octenal, (E)-2-hexenal, (E)-2-pentenal, and benzaldehyde, were generated by the oxidation of unsaturated fatty acids (UFAs), including linoleic acid (C18:2n-6), oleic acid (C18:1n-9), and linolenic acid (C18:3n-6) [33]. The levels of these aldehydes in the 8% group detected by GC-IMS above were significantly higher than in the NG. This suggests that the addition of AKM provided more precursors for the thermal processing and improved the ovarian flavor. In the study of smoked fish, Wang demonstrated that the hydroperoxide produced by the oxidation of UFAs was converted to aldehydes by β-cracking [34]. 2-propenal has a putrid and pungent odor, which was formed by the oxidation of TG during thermal processing [35]. During thermal processing, elevated temperatures catalyze the hydrolysis of TGs into glycerol and free fatty acids (e.g., linoleic acid, α- linolenic acid). The conjugated diene system in these fatty acids is highly sensitive to oxidation. Subsequent β- cracking of their hydroperoxide intermediates (ROOH) generates short-chain aldehydes such as 2-propenal [36]. The downregulation of TG in Group 3 could explain the significantly higher content of the undesirable odor component 2-propenal in the 8% group than in NG. Based on the upregulation of hexanal and heptanal in the 8% group, it could be seen that the downregulated FFA (17:1) acted as a precursor, which was oxidatively degraded during thermal processing, resulting in the production of hexanal and heptanal. In addition, the levels of nonanal and heptanal were higher in the 8% group than in the control group, while linoleic acid was abundant in AKM, and nonanal and heptanal could be generated from linoleic acid.

#### 3.4.2. Analysis of Amino Acid and Its Metabolites

The levels of L-methionine and L-norleucine were found to be downregulated in the EC group. FAAs are pivotal precursors engaged in Maillard reaction and Strecker degradation, and they are indispensable for the formation of distinctive aromas [37]. It has been demonstrated that sulphur-containing amino acids can be directly involved in the Maillard reaction to produce thiazoles, thiophenes, and numerous sulphur-containing compounds. Their aroma threshold is low, and they have strong volatility, making them an important component of the aroma of cooked aquatic products. L-methionine is a sulphur-containing amino acid that plays an instrumental role in the flavor profile of aquatic products. It can produce trimethylamine with a fishy smell and dimethyl trisulfide with a strong sulfur smell by Strecker degradation, which can also have a great impact on the flavor in the case of low level [38]. L-methionine was not detected in Group 2, presumably due to the relative lack of methionine in the peeled soy bean meal in NG feed. In contrast, AKM in the experimental group feed contained methionine, which resulted in higher levels of trimethylamine and dimethyl trisulfide in the 8% group than NG, thus enhancing the fishy smell of the ovaries. From the perspective of taste, L-methionine is a bitter amino acid, and its downregulation can weaken the bitterness of ovaries. Additionally, L-norleucine was also found to be downregulated, which generated glutaraldehyde during thermal processing. We speculated that the increase in glutaraldehyde content in the 8% group was related to the decrease in L-norleucine content in the EC group. Pentanal has the aroma of fruit, which has an important contribution to the aroma of *E. sinensis* ovaries. 5-hydroxytryptophan (5-HTP) is a metabolite of tryptophan. During thermal processing, the bitter tryptophan is degraded to form 5-HTP, resulting in an increase in the content of 5-HTP.

A total of 64 small peptides were detected in the EC group, of which 37 were upregulated and 27 were downregulated. Tyr-Gln-Val-Arg and Pro-Arg-Ala were most strongly upregulated and downregulated the most, respectively. Macromolecular peptides are rapidly degraded into small peptides, FAAs, and other flavor compounds during thermal processing, resulting in changes in the content of small peptides. They are essential for the formation of unique flavor properties. Among them, Val-Asp, Lys-His, Lys-Gly, Lys-Ile-Asp, His-Lys-Ser, Ile-Tyr-Asp, and Thr-Asp-Trp were upregulated, and these dipeptides or tripeptides containing Asp and Lys showed umami taste [39]. Anserine (β-alanyl-1-methyl L-histidine) and Glu-Phe were downregulated. Anserine is an imidazole dipeptide abundant in meat, which can reduce bitterness and enhance umami flavor. Moreover Glu-Phe can also significantly enhance umami flavor [40]. Its downregulation may reduce the umami of ovarian.

### 3.5. Analysis of Taste Related Metabolites Based on Group 4 (EC vs. NC)

The screening thresholds of VIP ≥ 1 and *p* value < 0.05 were employed, and some key taste compounds were screened and are displayed in Table 2.

Histidine, D-phenylalanine, 2-phenylglycine, and theanine were upregulated in the EC group, while L-norleucine and L-leucine were downregulated. Free amino acids, as some of the important flavor-presenting substances in crustaceans, give them a rich taste. Using the Bitter X ((https://mdl.shsmu.edu.cn/BitterX/) accessed on 10 October 2024) databases, histidine, D-phenylalanine, L-leucine, L-leucine, and DL phenylglycine were detected as bitter amino acids with low palatability. Theanine possesses a sweet and umami taste and has the capacity to effectively inhibit bitterness and enhance sweetness. Theanine is a beneficial feed additive, which increases the proportion of edible parts, immunity, antioxidant capacity, and gut microbiota abundance of female *E. sinensis* [41]. The taste of the ovaries is influenced by the content of flavor compounds. These flavor compounds in the ovaries interact with each other and jointly affect the taste of the ovaries. In general, the content of bitter substances in ovaries is low, which is not enough to cause the increase in bitterness. Moreover, the main taste of ovaries is umami, and the bitter taste is not easy to be felt.

Eight nucleotides and their metabolites were detected in the EC group. Among them, 5′-deoxy-5′-fluoradenosine, stavudine, cytarabine, and dihydrozearin nucleosides were upregulated, while 2′-O-methylguanosine, 2-methylguanosine, 1-methylguanosine, and nicotinamide nucleosides were downregulated. Cytarabine is a metabolite of the bitter hypoxanthine. Glycocholic acid showed an upward trend in the EC group. Glycocholic acid is a bile acid (BA), a natural ingredient that gives foods a bitter taste [42]. It is synthesized from cholesterol and is necessary for crustaceans such as shrimp and crabs to synthesize molting hormones. Unlike vertebrates, whether crustaceans can synthesize BAs has not been confirmed, and it needs to be obtained from feed. The upregulation of glycocholic acid in the EC group may enhance the bitterness of *E. sinensis* ovaries and have an adverse effect on their flavor. In addition, BAs may also contribute to the bitter flavor of *E. sinensis* hepatopancreas by promoting lipid metabolism, thereby inhibiting β-carotene uptake and enhancing amino acid metabolism [42]. Ethyl salicylate was found to be upregulated in organic acids and their derivatives. Obtained from the reaction of ethanol with salicylic acid, ethyl salicylate was an important flavor compound often used as a component in floral and fruity flavors. In addition, a water-soluble vitamin, biotin (vitamin H), was detected in the experimental group, which is a precursor substance of n-butanol. The lower level of n-butanol in the 8% group compared to the NG group may be related to the downregulation of biotin content.

The autoxidation of PUFA or the action of certain enzymes yields various oxylipins [43]. Oxylipins can cause browning, darkening to varying degrees, and bitterness [44]. Three types of oxylipins were detected, all of which were upregulated. Among them, 10-hydroxystearic acid has been shown to produce a bitter taste. Additionally, two types of free fatty acids, including α-linolenic acid (C18:3) and myristic acid (C14:0), were detected, all of which demonstrated an upward trend. Through database analysis, it was determined that all two free fatty acids were classified as bitter compounds. 10-hydroxystearic acid has been identified as an oxidation product of oleic acid (C18:1n9). Lipids containing C18:1n9, such as PE (O-18:3_18:1), were upregulated in the ER group. Concurrently, lipids containing two free fatty acids, α-linolenic acid, and myristic acid, such as PE (P-18:3_22:6) and PC (14:0_20:4), were also upregulated. After thermal processing, it ultimately led to upregulation of 10-hydroxystearic acid, α-linolenic acid, and myristic acid, resulting in bitterness. Consequently, the taste of the ovaries underwent a corresponding change in accordance with the alterations in the content of these flavor compounds.

## 4. Conclusions

This study indicates that crabs fed an 8% concentration of AKM exhibit the best ovarian flavor quality at the current addition level. AKM primarily influences the metabolism of glycerophospholipids, linoleic acid, and α-linolenic acid in the ovaries, which affects the production of precursor volatile flavor compounds and enhances flavor quality during thermal processing. AKM has a rich variety of nutrients, which systematically impacts the physiological metabolism of *E. sinensis*, resulting in comprehensive effects. In addition, the physiological metabolism and thermal processing processes can also create causal cascade effects that ultimately influence the flavor quality of *E. sinensis* ovaries. However, the content of substances that contribute to the ovarian flavor quality is not clear, so the key substances can be quantitatively analyzed later. In the future, response surface methodology (RSM) and other optimization methods can be used to systematically determine the ideal combination of AKM concentration and heat treatment parameters. At the same time, sensory evaluation experiments can be combined to validate the impact of changes in flavor compound content on flavor. The findings of this work provide a scientific basis for the development of functional feeds and high-quality *E. sinensis* farming.

## Figures and Tables

**Table 1:** Formulations of the experimental diets (%).

Ingredients	NG	PG	2%	4%	6%	8%
Peeled soybean meal (46%)	12.00	6.00	10.50	9.00	7.50	6.00
Soy protein concentrate	4.00	0.00	3.00	2.00	1.00	0.00
Fermented soybean meal	13.00	13.00	13.00	13.00	13.00	13.00
Fish meal	12.00	20.00	12.00	12.00	12.00	12.00
Rapeseed meal (36%)	8.00	8.00	8.00	8.00	8.00	8.00
Peanut meal	7.00	7.00	7.00	7.00	7.00	7.00
Chicken meal	8.00	7.00	8.00	8.00	8.00	8.00
Antarctic krill meal	0.00	0.00	2.00	4.00	6.00	8.00
Flour	17.20	20.60	18.20	19.20	20.20	21.20
Gluten meal	2.50	2.50	2.50	2.50	2.50	2.50
Bentonite	1.00	1.00	1.00	1.00	1.00	1.00
Penaeus polymorpha	1.20	1.20	1.20	1.20	1.20	1.20
Vitamin premix	1.50	1.50	1.50	1.50	1.50	1.50
Calcium dihydrogen phosphate	1.50	1.50	1.50	1.50	1.50	1.50
Choline chloride (60%)	0.60	0.60	0.60	0.60	0.60	0.60
Vitamin C (35%)	0.15	0.15	0.15	0.15	0.15	0.15
Vitamin E (50%)	0.05	0.05	0.05	0.05	0.05	0.05
Phospholipid oil	2.00	2.00	2.00	2.00	2.00	2.00
Refined fish oil	4.60	4.60	4.60	4.60	4.60	4.60
Soybean oil	4.60	4.20	4.10	3.60	3.10	2.60
Total (%)	100.00	100.00	100.00	100.00	100.00	100.00
Theoretical protein level (%)	38.53	38.52	38.54	38.55	38.56	38.57
Theoretical total fat level (%)	14.00	14.02	13.99	13.99	13.98	13.97
Theoretical energy value (MJ/kg)	19.11	19.06	19.10	19.09	19.08	19.06
Astaxanthin (mg/kg)	0.00	0.00	2.30	4.60	6.90	9.20
Lysine	2.14	2.19	2.14	2.15	2.15	2.15
Methionine	0.67	0.75	0.69	0.71	0.73	0.74

Vitamin premix (per kg diet): vitamin A, 13125 IU; vitamin D3, 5250 IU; vitamin E, 140 IU; vitamin K_3_, 14 mg; vitamin B_1_, 21 mg; vitamin B_2_, 33.6 mg; vitamin B_6_, 28.7 mg; vitamin B_12_, 0.07 mg; biotin, 0.28 mg; D-calcium pantothenate, 77 mg; folic acid, 7 mg; nicotinamide, 175 mg; vitamin C, 490 mg; inositol, 595 mg; ethoxyquin, 1.75 mg. NG: negative control group; and PG: positive control group.

**Table 2:** Changes in taste-related differential metabolite content from *E. sinensis* in Group 4.

Class	Compounds	Formula	Taste Attribute	FC	Type
Free amino acids	Histidine	C_6_H_9_N_3_O_2_	Bitter	2.131	Up
	L-theanine	C_7_H_14_N_2_O_3_	Sweet/Unami	1.294	Up
	D-phenylalanine	C_9_H_11_NO_2_	Bitter	1.060	Up
	2-phenylglycine	C_8_H_9_NO_2_	Bitter	8.541	Up
	L-leucine	C_6_H_13_NO_2_	Bitter	0.652	Down
	L-norleucine	C_6_H_13_NO_2_	Bitter	0.762	Down
Nucleotide and its metabolites	5′-deoxy-5′-fluoroadenosine	C_10_H_12_FN_5_O_3_	-	1.527	Up
	Stavudine	C_10_H_12_N_2_O_4_	-	1.498	Up
	Arainosine	C_10_H_12_N_4_O_5_	Bitter	1.433	Up
	Dihydrozeatin riboside	C_15_H_23_N_5_O_5_	-	1.151	Up
	2′-O-methylguanosine	C_11_H_15_N_5_O_5_	Unami	0.514	Down
	2-methylguanosine	C_11_H_15_N_5_O_5_	Unami	0.630	Down
	1-methylguanosine	C_11_H_15_N_5_O_5_	Unami	0.630	Down
	Nicotinamide riboside chloride	C_11_H_15_CN_2_O_5_	-	0.696	Down
Organic acid and its derivatives	Streptamine 4-phosphate	C_6_H_15_N_2_O_7_P	-	2.645	Up
	3-methyl-2-oxovaleric acid	C_6_H_10_O_3_	Bitter	2.097	Up
	3-Furoic acid	C_5_H_4_O_3_	Sour	1.666	Up
	Ethylsalicylate	C_9_H_10_O_3_	Sweet/Bitter	1.239	Up
	Piperonylic acid	C_8_H_6_O_4_	Bitter	1.224	Up
	2′-Cytidylic acid	C_9_H_14_N_3_O_8_P	Bitter	0.468	Down
	2-hydroxyethanesulfonate	C_2_H_6_O_4_S	Sour	0.540	Down
	2-O-(alpha-D-glucosyl)-sn-glycerol 3-phosphate	C_9_H_19_O_11_P	-	0.595	Down
	N-acetyl-alpha-D-glucosamine 1-phosphate	C_8_H_16_NO_9_P	-	0.681	Down
Sterol ester	Glycocholic acid	C_26_H_43_NO_6_	Bitter	1.834	Up
Free fatty acids	α-linolenic acid	C_14_H_28_O_2_	Bitter	1.442	Up
	Myristic acid	C_14_H_28_O_2_	Bitter	1.381	Up
Oxylipins	10-hydroxystearic acid	C_18_H_36_O_3_	Bitter	1.445	Up

Group 4: EC vs. NC; NC: negative cooked ovary; EC: experimental cooked ovary; and FC: fold change.

## Data Availability

The original contributions presented in the study are included in the article, further inquiries can be directed to the corresponding author.

## References

[1] Zhang L., Zhang R., Jiang X., Wu X., Wang X. (2024). Dietary supplementation with synthetic astaxanthin and DHA interactively regulates physiological metabolism to improve the color and odor quality of ovaries in adult female *Eriocheir sinensis*. Food Chem..

[2] Pan J., Wu X.G., Zhao H.L., He J., Jiang X.D., Wang Y.P., Cheng Y.X. (2016). Effects of three feeding modes on the culture performance of adult pond-reared Chinese mitten crab (*Eriocheir sinensis*) during the second year culture. Freshw. Fish..

[3] Tang S., Wang J.J., Li Y., Malakar P.K., Zhao Y. (2024). Recent advances in the use of antarctic krill (*Euphausia superba*) as a sustainable source of high-quality protein: A comprehensive review. Trends Food Sci. Technol..

[4] Kaur K., Kortner T.M., Benitez-Santana T., Burri L. (2022). Effects of Antarctic krill products on feed intake, growth performance, fillet quality, and health in salmonids. Aquac. Nutr..

[5] Sun P., Zhang X., Ren X., Cao Z., Zhao Y., Man H., Li D. (2022). Effect of basic amino acid pretreatment on the quality of canned Antarctic krill. Food Bioprocess Technol..

[6] Wei Y., Chen H., Jia M., Zhou H., Zhang Y., Xu W., Zhang W., Mai K. (2019). Effects of dietary Antarctic krill *Euphausia superba* meal on growth performance and muscle quality of triploid rainbow trout *Oncorhynchus mykiss* farmed in sea water. Aquaculture.

[7] Tharaka K., Benitez-Santana T., Gunathilaka B.E., Kim M., Lee C., Shin J., Lee K. (2020). Evaluation of Antarctic krill (*Euphausia superba*) meal supplementation in diets for olive flounder (*Paralichthys olivaceus*). Aquac. Res..

[8] Torrecillas S., Montero D., Carvalho M., Benitez-Santana T., Izquierdo M. (2021). Replacement of fish meal by Antarctic krill meal in diets for European sea bass *Dicentrarchus labrax*: Growth performance, feed utilization and liver lipid metabolism. Aquaculture.

[9] Wang X.Y. (2020). Effects of Antarctic Krill Meal on Growth Performance, Protein Metabolism, and Antioxidant Capacity in Juvenile 649 Chinese Mitten Crab (*Eriocheir sinensis*). Master’s Thesis.

[10] Chen X., Luo J., Lou A., Wang Y., Yang D., Shen Q.W. (2021). Duck breast muscle proteins, free fatty acids and volatile compounds as affected by curing methods. Food Chem..

[11] Ahn H.-S., Yeom J., Yu J., Kwon Y.-I., Kim J.-H., Kim K. (2020). Convergence of plasma metabolomics and proteomics analysis to discover signatures of high-grade serous ovarian cancer. Cancers.

[12] Feng L., Wang S., Chen H., Sun J., Zhang N., Zhang H. (2025). Fatty acid composition and key aroma components of two different cold pressed sesame oils. J. Food Compos. Anal..

[13] Song J., Wang H., Wu X., Wang X., Shi W. (2019). The flavor of gonad and meat of female *Portunus Trituberculatus* cultured in indoor and outdoor. J. Food Biochem..

[14] Iglesias J., Gallardo J.M., Medina I. (2010). Determination of carbonyl compounds in fish species samples with solid-phase microextraction with on-fibre derivatization. Food Chem..

[15] Jeleń H., Gracka A. (2016). Characterization of aroma compounds: Structure, physico-chemical and sensory properties. Flavour: From Food to Perception.

[16] Liu D., Bai L., Feng X., Chen Y.P., Zhang D., Yao W., Zhang H., Chen G., Liu Y. (2020). Characterization of Jinhua ham aroma profiles in specific to aging time by gas chromatography-ion mobility spectrometry (GC-IMS). Meat Sci..

[17] Reineccius G., Peterson D. (2013). Principles of food flavor analysis. Instrumental Assessment of Food Sensory Quality.

[18] Nogueira M.S., Scolaro B., Milne G.L., Castro I.A. (2019). Oxidation products from omega-3 and omega-6 fatty acids during a simulated shelf life of edible oils. LWT-Food Sci. Technol..

[19] Song S., Zhang X., Hayat K., Liu P., Jia C., Xia S., Xiao Z., Tian H., Niu Y. (2011). Formation of the beef flavour precursors and their correlation with chemical parameters during the controlled thermal oxidation of tallow. Food Chem..

[20] Ambasankar K., Dayal J.S., Vasagam KP K., Sivaramakrishnan T., Sandeep K.P., Panigrahi A., Raja R.A., Burri L., Vijayan K.K. (2022). Growth, fatty acid composition, immune-related gene expression, histology and haematology indices of *Penaeus vannamei* fed graded levels of Antarctic krill meal at two different fishmeal concentrations. Aquaculture.

[21] Jin S., Yue D., Fu H., Jiang S., Xiong Y., Qiao H., Wu Y. (2022). Effects of dietary supplementation with 17β-estradiol and 17α-methyltestosterone on growth performance and gonadal development of the juvenile oriental river prawn (*Macrobrachium nipponense*). Aquac. Rep..

[22] Song D., Shi B., Ding L., Jin M., Sun P., Jiao L., Zhou Q. (2019). Regulation of dietary phospholipids on growth performance, antioxidant activities, phospholipid metabolism and vitellogenesis in prereproductive phase of female swimming crabs, *Portunus trituberculatus*. Aquaculture.

[23] Pizzini A., Lunger L., Demetz E., Hilbe R., Weiss G., Ebenbichler C., Tancevski I. (2017). The role of omega-3 fatty acids in reverse cholesterol transport: A review. Nutrients.

[24] Majumdar R., Barchi B., Turlapati S.A., Gagne M., Minocha R., Long S., Minocha S.C. (2016). Glutamate, ornithine, arginine, proline, and polyamine metabolic interactions: The pathway is regulated at the post-transcriptional level. Front. Plant Sci..

[25] Fuke S., Konosu S. (1991). Taste-active components in some foods: A review of Japanese research. Physiol. Behav..

[26] Qi C., Wang X., Han F., Jia Y., Lin Z., Wang C., Lu J., Yang L., Wang X., Li E. (2019). Arginine supplementation improves growth, antioxidant capacity, immunity and disease resistance of juvenile Chinese mitten crab, *Eriocheir sinensis*. Fish Shellfish. Immunol..

[27] Ismail I., Hwang Y.H., Joo S.T. (2020). Low-temperature and long-time heating regimes on non-volatile compound and taste traits of beef assessed by the electronic tongue system. Food Chem..

[28] Zhang L., Yin M., Zheng Y., Xu C.-H., Tao N.-P., Wu X., Wang X. (2021). Brackish water improves the taste quality in meat of adult male *Eriocheir sinensis* during the postharvest temporary rearing. Food Chem..

[29] Zhang L., Yin M., Zheng Y., Tao N.-P., Wu X., Wang X. (2021). Short-term rearing in brackish water regulates the taste-related metabolites of abdomen muscle for adult male *Eriocheir sinensis*. LWT-Food Sci. Technol..

[30] Cui Z., Liu C., Rao W., Chen P., Lei K., Mai K., Zhang W. (2023). Dietary phospholipids improve growth performance and change the lipid composition and volatile flavor compound profiles in the muscle of abalone *Haliotis discus hannai* by affecting the glycerophospholipid metabolism. Aquac. Rep..

[31] Liu Q., Lin J., Zhao W., Lei M., Yang J., Bai W. (2023). The dynamic changes of flavors and UPLC-Q-Exactive-Orbitrap-MS based lipidomics in mackerel (*Scomberomorus niphonius*) during dry-cured processing. Food Res. Int..

[32] Ecker J., Liebisch G. (2014). Application of stable isotopes to investigate the metabolism of fatty acids, glycerophospholipid 564 and sphingolipid species. Prog. Lipid Res..

[33] Domínguez R., Pateiro M., Gagaoua M., Barba F.J., Zhang W., Lorenzo J.M. (2019). A comprehensive review on lipid 562 oxidation in meat and meat products. Antioxidants.

[34] Wang S., Adhikari K., Hung Y. (2017). Acceptability and preference drivers of freshly roasted peanuts. J. Food Sci..

[35] Vieira S.A., Zhang G., Decker E.A. (2017). Biological implications of lipid oxidation products. J. Am. Oil Chem. Soc..

[36] Shibata A., Uemura M., Hosokawa M., Miyashita K. (2015). Formation of acrolein in the autoxidation of triacylglycerols with different fatty acid compositions. J. Am. Oil Chem. Soc..

[37] Rivas-Cañedo A., Martínez-Onandi N., Gaya P., Nuñez M., Picon A. (2021). Effect of high-pressure processing and chemical composition on lipid oxidation, aminopeptidase activity and free amino acids of Serrano dry-cured ham. Meat Sci..

[38] Zhang S.-S., Guo S., Zheng Z.-J., Liu S.-J., Hou Y.-F., Ho C.-T., Bai N.-S. (2021). Characterization of volatiles in *Allium tenuissimum L.* flower by headspace-gas chromatography-olfactometry-mass spectrometry, odor activity values, and the omission and recombination experiments. LWT-Food Sci. Technol..

[39] Zhang Y., Venkitasamy C., Pan Z., Liu W., Zhao L. (2017). Novel umami ingredients: Umami peptides and their taste. J. Food Sci..

[40] Kajiya K., Arino M., Koshio A., Minami Y. (2023). Composition and taste of beef, pork, and duck meat and bioregulatory functions of imidazole dipeptides in meat. Sci. Rep..

[41] Yang M., Pan T., Li T., Duan G., Jiang H., Ling J. (2024). The effects of dietary L-theanine on the growth performance, non-specific immunity, antioxidant status, and intestinal microflora of female Chinese mitten crabs (*Eriocheir sinensis*). Aquac. Rep..

[42] Zhang L., Tao N.-P., Wu X., Wang X. (2022). Metabolomics of the hepatopancreas in Chinese mitten crabs (*Eriocheir sinensis*). Food Res. Int..

[43] Zhang R., Zhang L., Wu X., Wang X. (2024). Metabolomics analysis reveals bitter taste formation in off-season crab hepatopancreas marketed in June of the lunar calendar. J. Agric. Food Chem..

[44] Gigl M., Frank O., Barz J., Gabler A., Hegmanns C., Hofmann T. (2021). Identification and quantitation of reaction products from quinic acid, quinic acid lactone, and chlorogenic acid with Strecker aldehydes in roasted coffee. J. Agric. Food Chem..

